# QTL sequencing strategy to map genomic regions associated with resistance to ascochyta blight in chickpea

**DOI:** 10.1111/pbi.12964

**Published:** 2018-07-04

**Authors:** Amit Deokar, Mandeep Sagi, Ketema Daba, Bunyamin Tar'an

**Affiliations:** ^1^ Department of Plant Sciences University of Saskatchewan Saskatoon SK Canada

**Keywords:** ascochyta blight, chickpea, NGS‐based BSA, QTL, genomics, sequencing

## Abstract

Whole‐genome sequencing‐based bulked segregant analysis (BSA) for mapping quantitative trait loci (QTL) provides an efficient alternative approach to conventional QTL analysis as it significantly reduces the scale and cost of analysis with comparable power to QTL detection using full mapping population. We tested the application of next‐generation sequencing (NGS)‐based BSA approach for mapping QTLs for ascochyta blight resistance in chickpea using two recombinant inbred line populations CPR‐01 and CPR‐02. Eleven QTLs in CPR‐01 and six QTLs in CPR‐02 populations were mapped on chromosomes Ca1, Ca2, Ca4, Ca6 and Ca7. The QTLs identified in CPR‐01 using conventional biparental mapping approach were used to compare the efficiency of NGS‐based BSA in detecting QTLs for ascochyta blight resistance. The QTLs on chromosomes Ca1, Ca4, Ca6 and Ca7 overlapped with the QTLs previously detected in CPR‐01 using conventional QTL mapping method. The QTLs on chromosome Ca4 were detected in both populations and overlapped with the previously reported QTLs indicating conserved region for ascochyta blight resistance across different chickpea genotypes. Six candidate genes in the QTL regions identified using NGS‐based BSA on chromosomes Ca2 and Ca4 were validated for their association with ascochyta blight resistance in the CPR‐02 population. This study demonstrated the efficiency of NGS‐based BSA as a rapid and cost‐effective method to identify QTLs associated with ascochyta blight in chickpea.

## Introduction

Ascochyta blight caused by the necrotrophic fungus *Ascochyta rabiei* (Pass.) Labrousse is one of the most devastating diseases of chickpea (*Cicer arietinum* L.) worldwide. The disease incidence frequently occurs with high severity in areas with cool and wet growing conditions such as Canada, United States and some parts of Mediterranean countries (Sharma and Ghosh, 2016). Under favourable conditions, ascochyta blight can infect chickpea plants at any growth stage. However, the crop is more susceptible at flowering and podding stages causing substantial economic damage to the crop (Sharma *et al*., 2010). A significant decline in chickpea production in Canada and Australia in the past decade was resulted from heavy yield losses caused by ascochyta blight (Armstrong‐Cho *et al*., 2008; Bretag *et al*., 2008). At present, successful chickpea production in many areas depends on effective ascochyta blight management. Genotypes with complete resistance to ascochyta blight in chickpea are lacking. However, moderately resistant genotypes have been identified and used to develop cultivars with improved resistance. In areas where ascochyta blight infection is predominant, these cultivars were used along with fungicide applications to manage the disease. This strategy, however, is often ineffective when the conditions for *Ascochyta rabiei* infection are highly conducive. Therefore, continuing efforts to develop new cultivars with improved resistance to ascochyta blight is required to sustain chickpea production.

Resistance to ascochyta blight is polygenic and is often highly affected by environmental conditions (Armstrong‐Cho *et al*., 2008). Several QTLs associated with ascochyta blight resistance with low‐to‐moderate effects have been identified in chickpea. QTLs for resistant to *Ascochyta rabiei* pathotypes I, II and III were identified on linkage groups 2, 3, 4 and 6 (Aryamanesh *et al*., 2010; Cho *et al*., 2004; Taleei *et al*., 2009; Tar'an *et al*., 2007; Udupa and Baum, 2003). Apart from the pathotype‐specific QTLs, several other QTLs for ascochyta blight resistance were also identified in diverse genetic backgrounds (Anbessa *et al*., 2009; Daba *et al*., 2016; Flandez‐Galvez *et al*., 2003; Iruela *et al*., 2006; Millan *et al*., 2003; Sabbavarapu *et al*., 2013; Santra *et al*., 2000; Tekeoglu *et al*., 2002). The majority of these QTLs were identified using low‐density genetic maps, and, hence, the QTLs were mapped within large genomic interval containing hundreds of potential candidate genes. This limits the potential application of those QTLs for gene cloning and marker‐assisted selection in chickpea.

QTL mapping requires genotyping and phenotyping of a large number of progenies from biparental mapping population, which is time‐consuming and labour‐intensive. BSA has been used to overcome this issue by genotyping only lines with extreme phenotypes instead of a large number of individuals in a mapping population (Michelmore *et al*., 1991). BSA has been successfully used in finding several large effects QTLs using common molecular marker systems (Asnaghi *et al*., 2004; Gillman *et al*., 2011; Halldén *et al*., 1997). Recent advances in DNA sequencing technology have provided effective tools for genome‐wide single nucleotide polymorphism (SNP) marker discovery and genotyping, such as whole‐genome sequencing, which provides a large number of genome‐wide SNPs and other structural variants (Huang *et al*., 2009). However, whole‐genome sequencing of a large number of segregating population is still very expensive. A combined approach of whole‐genome sequencing and BSA has been found effective in term of cost and time to quickly identify genomic regions associated with the trait of interest (Liu *et al*., 2012; Takagi *et al*., 2013). In the NGS‐based BSA analysis, individuals with two extreme phenotypes (e.g. resistant and susceptible) from a segregating population are pooled separately and sequenced using NGS platforms, and, then, allele frequency in each pool is compared. In the majority of the genomic regions, allele frequency between the two bulks should be approximately equal, except in the regions associated with the phenotype (Magwene *et al*., 2011). Genomic regions with significant differential allele frequencies between the bulks reflect the association of the regions with the QTLs associated with the trait. After detecting the QTL, the confidence interval for its location is determined using appropriate statistical tests (Magwene *et al*., 2011; Takagi *et al*., 2013). The NGS‐based BSA approach has received much attention over the last few years due its efficiency for mapping quantitative traits, and increased accessibility and affordability of the NGS platforms. Furthermore, the availability of NGS‐based BSA analysis tools in the form of a standalone software package (e.g. QTL‐seq package), and R package (e.g. QTLseqr) has simplified the NGS‐based BSA analysis to a great extent (Mansfeld and Grumet, 2018; Takagi *et al*., 2013). NGS‐based BSA approach has been successfully used to map QTLs for various traits with different levels of genetic complexities from single gene to multiple major QTLs (Chen *et al*., 2017; Das *et al*., 2015, 2016; Illa‐Berenguer *et al*., 2015; Kaminski *et al*., 2015; Lu *et al*., 2014; Pandey *et al*., 2017; Singh *et al*., 2016; Takagi *et al*., 2013).

In this study, we identified genomic regions associated with ascochyta blight resistance using NGS‐based BSA approach in two recombinant inbred populations of chickpea. To examine the efficiency of NGS‐based BSA for mapping the QTLs for ascochyta blight resistance, the QTLs identified in the first population (CPR‐01) were compared to the previously identified QTLs in the same population using the entire population. The second population (CPR‐02) was evaluated for ascochyta blight reaction in multiyears under greenhouse and field conditions and was used for rapid mapping of QTLs for ascochyta blight resistance using NGS‐based BSA. The resistance sources used in this study were CDC Frontier and Amit. CDC Frontier was selected from progeny of a cross between FLIP 91‐22C and ICC 14912, whereas Amit is a selection from Bulgarian landrace. Based on the pedigree information, there is no common source of resistance between CDC Frontier and Amit. However, genetic analysis of ascochyta blight reaction in the F_1_ and F_2_ generations showed that the reaction to ascochyta blight in the populations derived from CDC Frontier and Amit was similar and possibly shared common QTLs for ascochyta blight resistance (Anbessa *et al*., 2009).

Genomic regions identified in this study can be used to narrow down the region to facilitate identification of the potential candidate genes for the disease resistance and to develop diagnostic markers to allow pyramiding multiple QTLs to enhance ascochyta blight resistance in chickpea.

## Results

### The response of the RIL populations to ascochyta blight infection and development of bulk segregants

Analyses of variance (ANOVA) of CPR‐02 showed significant effects of genotype, environment (year) and their interaction for ascochyta blight severity under both greenhouse and field conditions (Table S1). Broad‐sense heritability (H^2^) estimates ranged from 0.48 to 0.65 under greenhouse and field screenings. Similar observation of significant effects of genotype, environment (year) and their interaction for ascochyta blight severity was observed in CPR‐01 in 2012–2013 (Daba *et al*., 2016). The ascochyta blight disease scores among the RILs in the CPR‐01 ranged from 2.0 to 9.0. The resistant parent CDC Frontier had an overall mean disease score of 4.0 (ranged from 3.0 to 4.7), while the susceptible parent ICCV 96029 had a mean disease score of 7.5 (ranged from 7.3 to 8.0). Based on the ascochyta blight scores of the RILs, ten individuals with the lowest and highest disease scores were selected and pooled as resistant (CPR01‐RB) and susceptible (CPR01‐SB) bulks, respectively (Figure S1). The average disease score based on multiple field and greenhouse disease screenings of the CPR01‐RB bulk was 4.0 (ranged from 3.4 to 4.5), while the average disease score of the CPR01‐SB bulk was 7.7 (ranged from 7.5 to 8.0).

**Figure 1:**
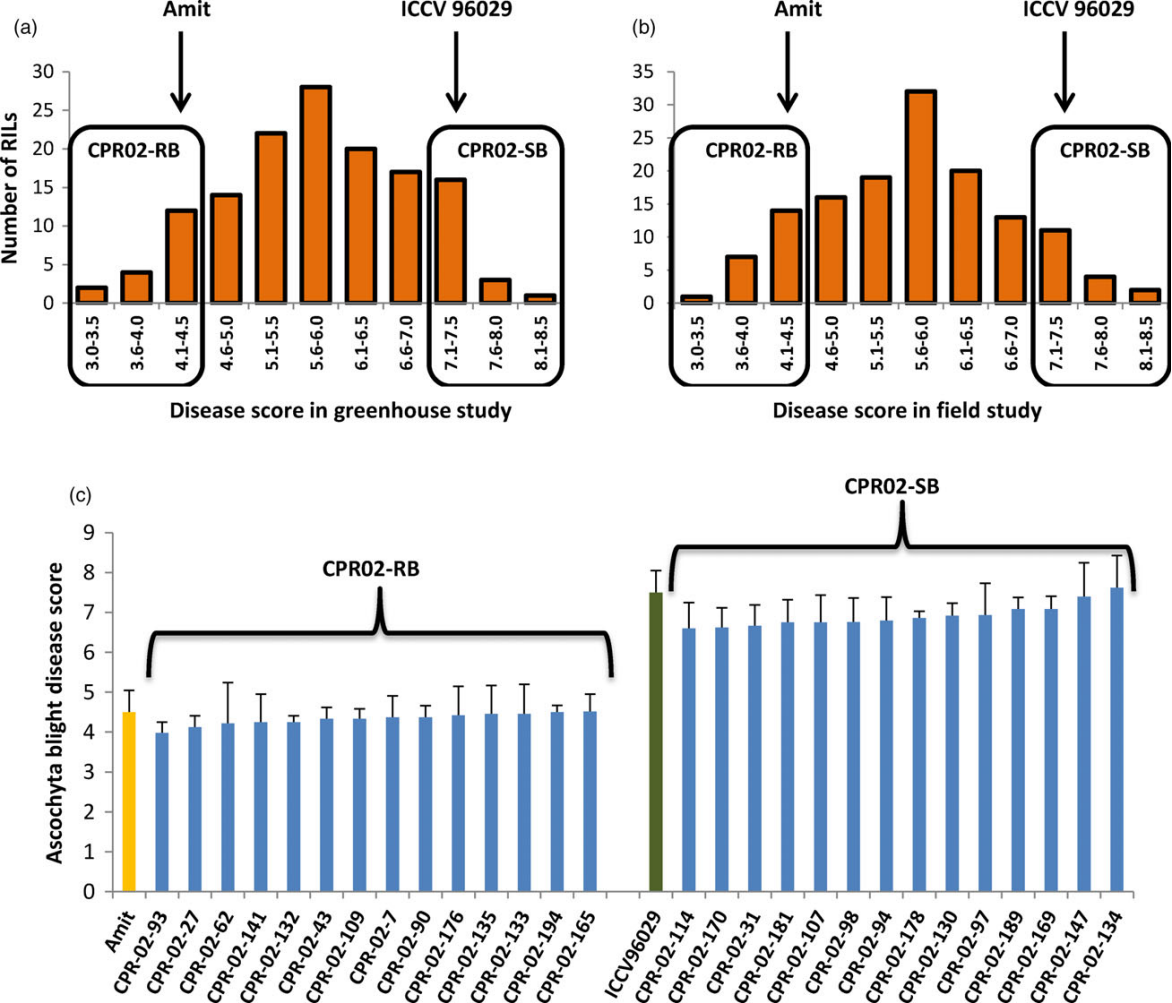
Frequency distribution of ascochyta blight disease scores and the selected RILs exhibiting extreme disease scores used to construct resistant and susceptible bulks. (a) Frequency distribution of disease score on 0–9 scales of the CPR‐02 population under greenhouse and (b) field conditions. Arrows show the mean score of the resistant (Amit) and the susceptible (ICCV 96029) parents. (c) Based on the disease score, 14 RILs exhibiting extreme disease response on both ends of the scale were selected to construct resistant (CPR02‐RB) and susceptible (CPR02‐SB) bulks. Mean disease scores of the resistant parent Amit and the susceptible parent ICCV 96029 are shown in yellow and green colour bars.

The disease scores of the CPR‐02 RILs in response to ascochyta blight ranged from 3.0 to 8.0 (Figure 1). The resistant parent Amit had an overall mean disease score of 4.5 (ranged from 4.0 to 4.8), while the susceptible parent ICCV 96029 had a mean disease score of 7.5 (ranged from 7.0 to 7.8). The frequency distribution of ascochyta blight scores in CPR‐01 and CPR‐02 populations followed a normal distribution pattern suggesting that the resistance to ascochyta blight disease is polygenic and is likely controlled by multiple QTLs. Based on the ascochyta blight scores of the CPR‐02 population, 14 individuals from each of the extreme ends of the phenotypic distribution were selected to form resistant (CPR02‐RB) and susceptible (CPR02‐SB) bulks, respectively (Figure 1). The average disease score of CPR02‐RB was 4.2 (ranged from 4.0 to 4.5), while CPR02‐SB was 7.4 (ranged from 6.6 to 7.6) as observed in multiple field and greenhouse screenings.

### Whole‐genome sequencing and mapping of resistant and susceptible bulks

Whole‐genome sequences of pooled DNAs along with the parental lines of both RIL populations (ICCV 96029 and Amit) were generated using paired‐end sequencing on Illumina Hiseq platform. CDC Frontier (one of the parents of the CPR‐01 population), for which the whole‐genome sequence is available, was used for mapping CPR01‐RB and CPR01‐SB reads. The whole‐genome sequencing generated 53.9 and 47.2 m paired‐end (PE) reads for ICCV 96029 and Amit, respectively, which provided on average 16.9X and 14.7X coverage of the chickpea genome, respectively.

Sequencing of CPR‐01‐resistant (CPR01‐RB) and susceptible (CPR01‐SB) bulks generated 52.1 and 56.7 m PE reads, respectively. The sequence reads of the bulked samples provided on average 18.9 X coverage for CPR01‐RB and 20.5 X coverage for CPR01‐SB of the chickpea genome. On average, 91.1% of the reads from both bulked samples were mapped to the chickpea genome.

Sequencing of the CPR‐02 bulked samples generated 48.4 and 55.5 m PE reads for the CPR02‐RB and CPR02‐SB, respectively. The sequence reads of the bulked samples provided on average 17.0 X for CPR02‐RB and 20.8 X for CPR02‐SB coverage of the chickpea genome. On average, 91.7% of the reads from both parents and the bulked samples were mapped to the chickpea genome. Alignment of the reads from ICCV 96029 onto CDC Frontier reference genome assembly (version 2.6.3) identified 566 949 SNPs and 129 987 InDels. Based on the SNP information, the reference genome of ICCV 96029 was generated by replacing the reference bases in the CDC Frontier genome assembly with the alternative bases. This pseudo‐reference genome assembly of ICCV 96029 was used for mapping the reads from CPR02‐RB and CPR02‐SB. In total, 501 021 (CPR02‐RB) and 502 633 (CPR02‐SB) SNPs were identified after their alignments with the ICCV 96029 reference genome.

Variant calling resulted in 77 938 SNPs in CPR‐01 bulks when they were mapped to the CDC Frontier (V2.6.3) reference genome. In CPR‐02 bulks, 106 907 SNPs were identified when they were mapped to the ICCV 96029 pseudo‐reference genome assembly. The SNPs were subjected to NGS‐based BSA analysis after filtering the SNPs that are over or under‐represented in both resistant and susceptible bulks.

### NGS‐based BSA analysis

**Figure 2:**
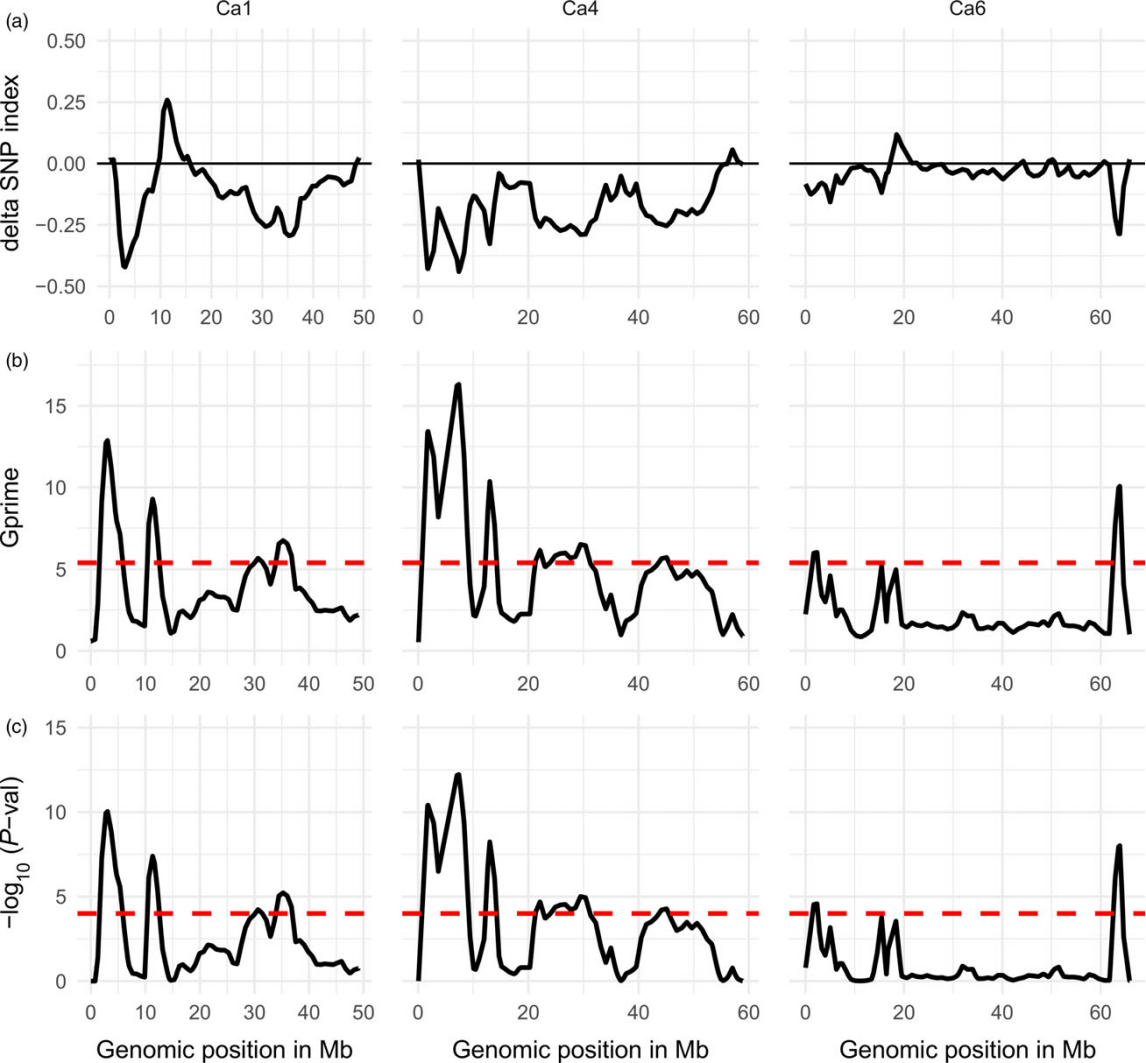
Quantitative trait loci for ascochyta blight resistance in chickpea on chromosomes Ca1, Ca4 and Ca6 identified in CPR‐01 (ICCV 96029 X CDC Frontier) using NGS‐based BSA. Distribution of the delta SNP index (a), G′ value (b) and –log10 *P*‐value (c) calculated with a 1‐Mb sliding window using tricube smoothing kernel. The *Y*‐axis represents delta SNP index, G′ value and –log10 *P*‐value in figure subsection a, b and c, respectively. The *X*‐axis represents the position of chromosomes in Mb based on the CDC Frontier genome assembly v 2.6.3. The dotted red line in b and c indicates the significant threshold for FDR = 0.001 and genomic region where the G′ or –log10 *P*‐value crosses the threshold value was considered as significant QTL. The positive delta SNP index indicates that the nonreference allele (ICCV 96029) contributes to the trait, whereas the negative delta SNP index shows that reference alleles (CDC Frontier) contribute to the trait. Only chromosomes with significant QTL regions are shown.

The SNP index and G statistics value of the individual SNPs were calculated as described by Magwene *et al*. (2011) and Takagi *et al*. (2013). The tricube‐smoothed delta SNP index and G value (G′ value) were calculated within a window size of 1.0 Mb genomic region and were plotted against all eight chickpea chromosomes (Figure 2). Significant thresholds (*P*‐values) were estimated from the null distribution of G′ assuming there is no QTL linked to the SNP (Magwene *et al*., 2011; Yang *et al*., 2013). The tricube‐smoothed G′ values of the CPR01 analysis showed significant G′ peaks on chromosomes Ca1, Ca4 and Ca6 above the FDR (q) of 0.001 suggesting that these G′ peak regions most likely contain the QTL for ascochyta blight resistance in CPR01 (Table S2). Chromosome Ca1 has four significant QTLs and were named as CPR01‐qAB1.1, CPR01‐qAB1.2, CPR01‐qAB1.3 and CPR01‐qAB1.4. Similarly, chromosome Ca4 has five significant QTLs as CPR01‐qAB4.1, CPR01‐qAB4.2, CPR01‐qAB4.3, CPR01‐qAB4.4 and CPR01‐qAB4.5. Two significant QTLs were identified on chromosome Ca6 as CPR01‐qAB6.1 and CPR01‐qAB6.2. The direction of the delta SNP value indicated that all of the QTLs in CPR‐01, except CPR01‐qAB1.2, originated from the resistant parent CDC Frontier, whereas the CPR01‐qAB1.2 was contributed by the ICCV 96029 parent (Table S2). Among all the CPR‐01 QTLs, CPR01‐qAB1.1 and CPR01‐qAB4.1 showed the highest G′ peaks indicating major QTLs for ascochyta blight resistance. The regions covered by the significant QTLs varied from 1.3 Mb (CPR01‐qAB6.1) to 7.0 Mb (CPR01‐qAB4.4).

**Figure 3:**
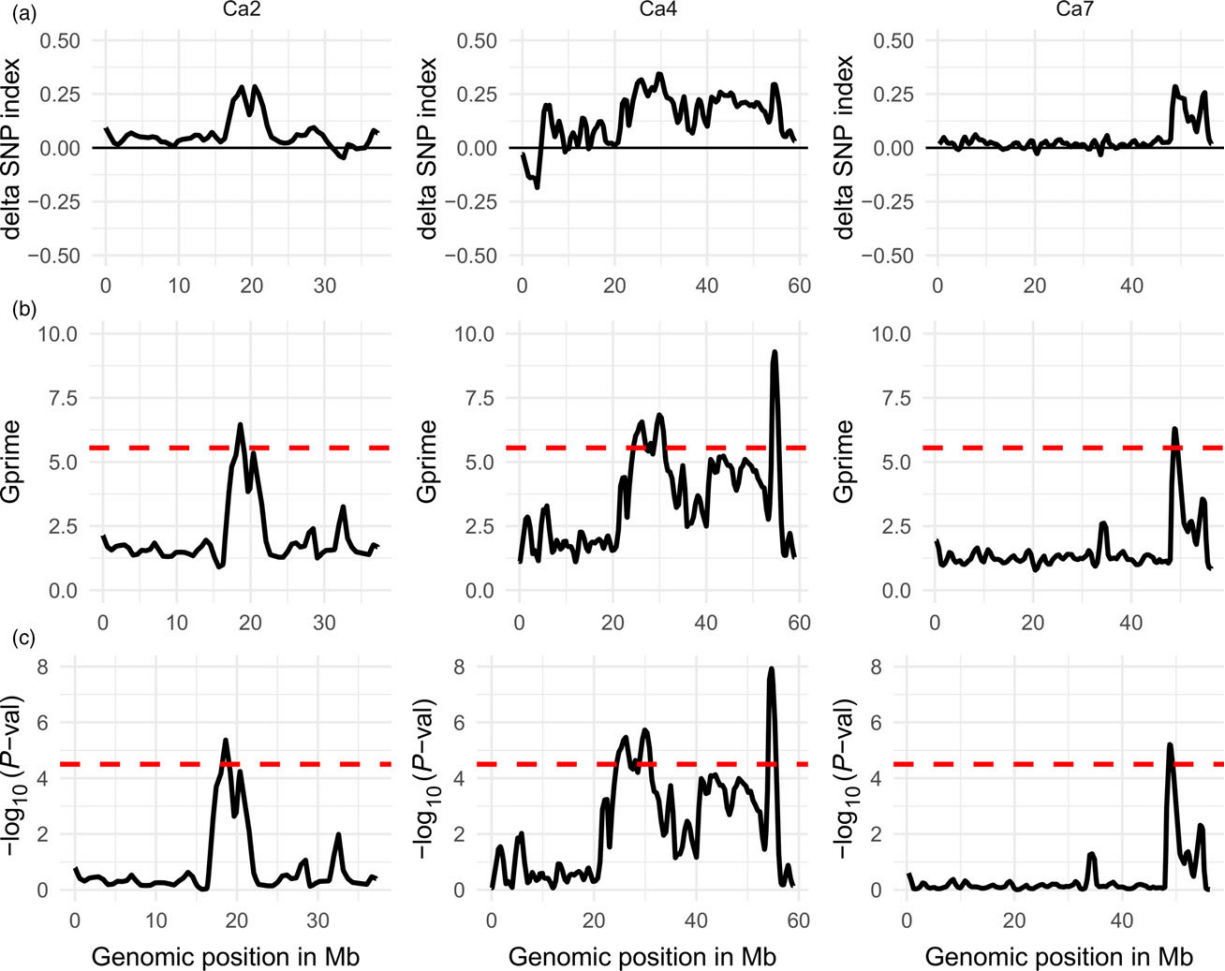
Quantitative trait loci for ascochyta blight resistance in chickpea on chromosomes Ca2, Ca4 and Ca7 identified in CPR‐02 (ICCV96029 X Amit) using NGS‐based BSA. Distribution of the delta SNP index (a), G′ value (b) and –log10 *P*‐value (c) calculated with a 1‐Mb sliding window using tricube smoothing kernel. The *Y*‐axis represents delta SNP index, G′ value and – log_10_ *P*‐values in subsection a, b and c, respectively. The *X*‐axis represents the position of chromosomes in Mb based on the CDC Frontier genome assembly V2.6.3. The dotted red line in (b and c) shows the significance threshold for FDR = 0.001, and genomic region where the G′ or –log10 *P*‐value crosses the threshold value was considered as significant QTL. Of eight chickpea chromosomes, only three chromosomes with significant QTL regions are shown.

The tricube‐smoothed G′ values of the CPR‐02 analysis showed significant G′ peaks on chromosomes Ca2, Ca4 and Ca7 above the FDR (q) of 0.001 (Figure 3). Chromosome Ca2 has one significant QTL CPR02‐qAB2.1. Similarly, chromosome Ca4 has four significant QTLs (CPR02‐qAB4.1, CPR02‐qAB4.2, CPR02‐qAB4.3 and CPR02‐qAB4.4). One significant QTL (CPR02‐qAB7.1) was identified on chromosome Ca7. The direction of the delta SNP value indicated that all of the QTLs in CPR‐02 originated from the resistant parent Amit. The genomic region covered by the significant QTL varies from 0.2 Mb (CPR02‐qAB7.1) on chromosome Ca7 to 2.71 Mb (CPR02‐qAB4.1) on chromosome Ca4. In the QTL CPR02‐qAB2.1 region, the major G′ peak exhibited a subpeak; however, it falls below the FDR (q) of 0.001. The genomic region on chromosome Ca4 starts from 24.3 to 31.0 Mb and contains three QTLs (CPR02‐qAB4.1, CPR02‐qAB4.2 and CPR02‐qAB4.3). This region showed two clear G′ peaks and one subpeak between the two peaks; this suggested the possibility of multiple QTLs linked in coupling phase with all the resistant alleles were derived from the resistant parent Amit.

### Common genomic regions associated with ascochyta blight resistance in Amit and CDC Frontier

**Figure 4:**
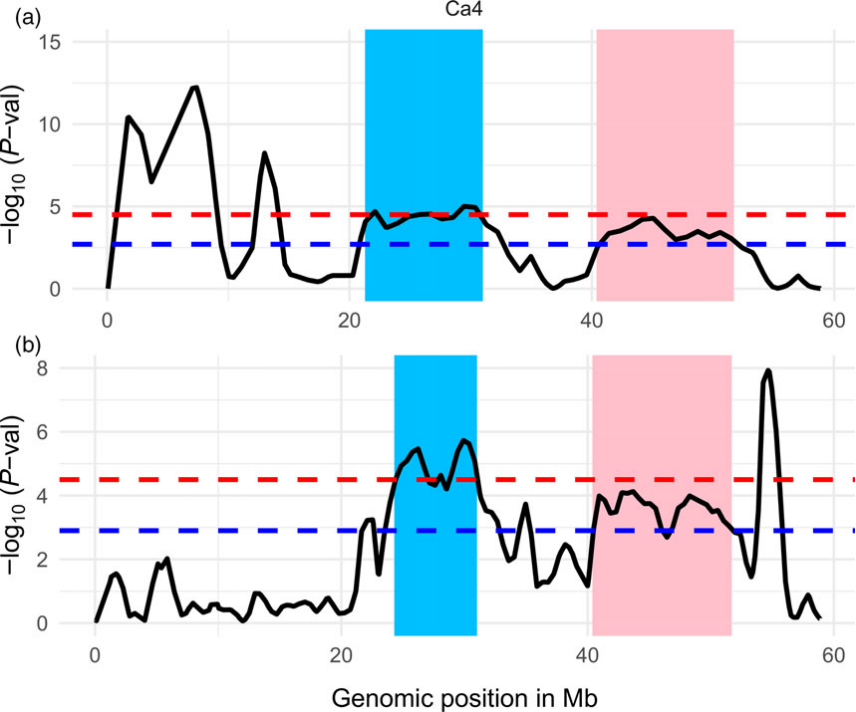
Common genomic regions associated with resistance to ascochyta blight in two chickpea cultivars CDC Frontier and Amit. Two genomic segments on chromosome Ca4 (highlighted with blue and pink) identified in CPR‐01 (a) and CPR‐02 (b) contain significant QTLs with FDR of 0.001 and 0.01 as shown by the dashed line in red and blue colours.

CDC Frontier and Amit are two moderately resistant cultivars and have distinct genetic backgrounds. However, QTLs on chromosome Ca4 were identified in common genomic regions in both populations (Figure 4). The genomic region from 24.3 to 31.0 Mb contains CPR01‐qAB4.4 in CPR‐01 and a cluster of QTLs (CPR02‐qAB4.1, CPR02‐qAB4.2 and CPR02‐qAB4.3) in CPR‐02. Another genomic region between 43.7 and 45.3 Mb on the same chromosome also contains CPR01‐qAB4.5 which overlapped with two QTLs identified in CPR‐02 though with a slightly lower significant level (FDR [q] of 0.01). All the QTLs in the Ca4 have the resistant parents CDC Frontier and Amit as the sources of the resistance. This suggests that these two cultivars share common genomic regions associated with ascochyta blight resistance on chromosome Ca4.

### Comparison of QTLs identified by conventional biparental and NGS‐based BSA mapping approaches

The CPR‐01 mapping population was originally genotyped using restriction site‐associated DNA sequencing genotyping‐by‐sequencing (RAD‐seq GBS) method, and a high‐density linkage map was constructed using 30 225 SNP markers representing 1336 recombination bins (Deokar *et al*., 2014). Eight QTLs which individually explained 9%–19% of the phenotypic variations were identified using conventional QTL mapping approach (Daba *et al*., 2016). Five QTLs identified in this study overlapped with four QTLs identified using the entire CPR‐01 population through conventional QTL analysis (Table 1). CPR01‐qAB1.1 identified in this study is the same as *qAB1.1* reported earlier in CPR‐01 (Daba *et al*., 2016). Two QTLs on chromosome Ca4 CPR01‐qAB4.1 and CPR01‐qAB4.2 also overlapped with a single QTL *qAB4*.1 identified by the conventional biparental mapping method. The *qAB4.1* mapped at 6.9‐13.4 Mb, whereas the CPR01‐qAB4.1 and CPR01‐qAB4.2 mapped at 1.7‐8.9 and 12.2‐14 Mb on Ca4. These results showed that NGS‐based BSA was able to detect two linked QTLs that were initially identified as a single QTL using conventional biparental mapping.

**Table 1:** Comparison of QTLs for resistance to ascochyta blight identified using NGS‐based BSA approach and conventional full population QTL mapping

NGS‐based BSA^a^	QTL^b^	Chromosome	Interval (cM) QTL^b^	Interval (Mb) QTL^b^	P.V.E (%) QTL^b^	Interval^c^ (Mb) NGS‐based BSA^a^
CPR01‐qAB1.1	*qtlAb‐1.1*	1	18.8–21.1	3.5–5	13	2–5.3
CPR01‐qAB1.2		1				10.6–12.4
CPR01‐qAB1.3		1				30.2–31.4
CPR01‐qAB1.4		1				34–36.8
	*qtlAb‐2.1*	2	41.4–47.2	15.3–17.1	14	
	*qtlAb‐3.1*	3	8.6–23.5	4.1–9.6	15	
				17.8–31.2		
				42.8–61.9		
CPR01‐qAB4.1	*qtlAb‐4.1*	4	15.5–36.2	6.9–13.4	17	1.7–8.9
CPR01‐qAB4.2		4				12.2–14
CPR01‐qAB4.3		4				21.3–22.7
CPR01‐qAB4.4		4				24.1–31.1
CPR01‐qAB4.5		4				43.8–45.4
CPR01‐qAB6.1	*qtlAb‐6.1*	6	26.9–52.7	0.6–5.1	19	1.5–2.3
CPR01‐qAB6.2		6		63.5–64.2		62.9–64.2
CPR01‐qAB7.1	*qtlAb‐7.1*	7	45.0–57.7	1.8–6.6	10	3.4–4.8
	*qtlAb‐8.1*	8	72.0–75.8	0–1	12	
	*qtlAb‐8.2*	8	1.6–14.4	7.8–13.4	16	
	*qtlAb‐8.3*	8	53.8–54.3	13.4–13.8	9	

a QTLs identified in CPR01 using NGS‐based BSA approach.

b QTLs identified in CPR01 using full population QTL mapping approach (Daba *et al*., 2016).

c Based on CDC Frontier reference genome assembly version 2.6.3

P.V.E (%): Percentage of phenotypic variance explained (PVE) by each QTL.

Two QTLs on chromosome Ca6 CPR01‐qAB6.1 and CPR01‐qAB6.2 overlapped with a single QTL *qAB6.1*. However, this is likely because of an error in genome assembly; as the physical location of the QTL in the reference genome assembly, CDC Frontier (v2.6.3) splits into two genome segments 0.6–5.1 Mb and 63.5–64.2 Mb on chromosome Ca6, whereas in the first draft of chickpea genome assembly, the QTL *qAB6.1* was mapped as a single genomic segment from 10.2 to 17.2 Mb on the same chromosome. The common QTLs identified using NGS‐based BSA and conventional biparental QTL mapping population individually explained 13%–19% of the phenotypic variations, whereas some QTLs (explained 9%–14% of the phenotypic variations) identified in the CPR‐01 using conventional full population QTL mapping approach were not detected in the NGS‐based BSA analysis (Table 1). Overall, the results showed that QTLs with moderate to relatively large effects could be identified using the NGS‐based BSA approach with marginal population size.

### SNP marker development and QTL mapping

The competitive allele‐specific PCR (KASP™) genotyping assay was developed for the SNPs within the potential candidate genes identified in the QTL intervals. Nonsynonymous SNPs within the potential candidate genes were selected for KASP assay design. Two KASP assays for two SNPs, each from chromosomes Ca2 and Ca4 that are not associated with ascochyta blight response, were designed as checks for the QTL analysis. Eight KASP markers were developed and used for genotyping CPR‐02 RIL population. In the CPR02‐qAB2.1 region, KASP markers for two NBS‐LRR genes (Ca30037 and Ca30038) and an ankyrin repeat domain‐containing protein gene (Ca30034) were developed. Three KASP markers, each for ethylene receptor 2 (Ca12910), photoperiod‐independent early flowering 1 (Ca13027) and flowering locus D (Ca14012), were designed as representative SNPs within the ascochyta blight QTLs on chromosome Ca4. Primer sequences of all KASP markers are provided in Table S3. All the primers could distinguish SNP alleles from Amit and ICCV 96029 in the CPR‐02 RILs.

We used single marker analysis of variance to identify the QTLs in CPR‐02 population. All the three candidate gene‐based SNP markers on chromosome Ca2 within the CPR02‐qAB2.1 interval showed significant association with ascochyta blight resistance with the *P*‐value <0.05 and accounted for 4%–6% of the phenotypic variation (Table 2). SNPs within the ascochyta blight QTLs on chromosome Ca4 were also showed significant association with the *P*‐value of <0.0001 and accounted for 6%–14% of the phenotypic variation for ascochyta blight.

**Table 2:** Validation of SNP markers associated with resistance to ascochyta blight resistance using the single marker ANOVA method

Chr*	SNP marker	QTL	*P*‐value	% R2	Annotation
Ca2	Ca2v2.6p18233152_G/A	CPR02‐qAB2.1	*<0.05*	5.4	Ankyrin repeat domain‐containing protein
Ca2	Ca2v2.6p18250143_T/A	CPR02‐qAB2.1	*<0.05*	5.4	TNL class (NBS‐LRR)
Ca2	Ca2v2.6p18266481_A/C	CPR02‐qAB2.1	<0.001	6.0	TNL class (NBS‐LRR)
Ca2	Ca2v2.6p28572458_G/A	Unlinked marker	NS	–	Glycosylphosphatidylinositol anchor attachment 1 protein
Ca4	Ca4v2.6p26669292_T/G	CPR02‐qAB4.1	<0.001	12.1	Ethylene receptor 2
Ca4	Ca4v2.6p28791114_G/C	CPR02‐qAB4.2	<0.0001	13.5	Photoperiod‐independent early flowering 1
Ca4	Ca4v2.6p43806808_A/G	CPR02‐qAB4.5	<0.0001	12.5	Flowering locus D (FLD)
Ca4	Ca4v2.6p904185_A/C	Unlinked marker	NS	–	Succinyl‐CoA ligase subunit beta

a Chromosome (Chr.) positions are based on the CDC Frontier reference genome assembly v2.6.3

Unlinked SNP marker: SNP markers that were not found associated with ascochyta blight in our NGS‐based BSA analysis and used as a negative check in a marker–trait association study.

*P*‐value: *P*‐value greater than 0.05 was regarded as a non‐significant (NS) association, whereas *P* value of less than 0.05 was considered as significant marker‐trait association.% R2: R2 value represents the percentage of phenotypic variance for ascochyta blight resistance explained by each significant SNP.

## Discussion

In chickpea, NGS‐based BSA has successfully been used to map genetic loci associated with seed weight (Das *et al*., 2015; Singh *et al*., 2016), pod number (Das *et al*., 2016) and root traits (Singh *et al*., 2016). Most of these traits have medium‐to‐high heritability and QTLs with large effects were detected using conventional biparental QTL mapping (Bajaj *et al*., 2015). In the present study, we used NGS‐based BSA approach to identify genomic regions associated with ascochyta blight resistance. Ascochyta blight resistance is a complex trait with low‐to‐moderate heritability and is highly influenced by environmental conditions. Two RIL populations (CPR‐01 and CPR‐02) were screened under controlled conditions in the greenhouse and under field conditions for multiple years and locations to improve the reproducibility of the disease scores. Chickpea response to ascochyta blight is difficult to quantify accurately because the resistance is expressed on a continuous distribution. In the majority of the screenings, the disease response was measured using nonparametric 0–5 or 0–9 rating scales based on the visible symptoms of the disease such as the lesion on leaves, apical stem, stem and on all above ground plant parts, defoliation, breaking and drying of branches (Chen *et al*., 2004; Reddy and Singh, 1984). The large variation in the score values for ascochyta blight response especially at the middle of the scale was observed in the 0–9 scale (Tivoli *et al*., 2006), which affects the conventional QTL mapping as the QTL mapping needs precise phenotypic values of each line in a population. However, the 0–9 scale was more precise at the extremes of the scale than the middle scale, which helps in identification of individuals with extreme phenotypes which is a key step in the BSA. Using 0–9 scale to identify lines with extreme phenotypes (resistant and susceptible) and NGS‐based BSA greatly simplified and expedited the QTL mapping process of ascochyta blight in chickpea as demonstrated in this study.

Complete resistance to *Ascochyta rabiei* has not been identified in cultivated chickpeas; however, moderate resistance in some genotypes has been identified in several chickpea accessions (Sharma and Ghosh, 2016; Tar'an *et al*., 2007). Among the kabuli chickpea cultivars that are well adapted to the Canadian growing conditions, CDC Frontier and Amit have moderate resistance to ascochyta blight (Anbessa *et al*., 2009; Tar'an *et al*., 2007). However, precise genomic regions associated with the response to ascochyta blight in CDC Frontier and Amit are not clear. RILs produced by crossing a highly susceptible genotype ICCV 96029 with partially resistant genotype CDC Frontier (CPR‐01) and Amit (CPR‐02) were used for mapping QTLs associated with ascochyta blight resistance (Anbessa *et al*., 2009). In the present study using the whole‐genome sequencing and BSA approach, we identified 11 QTLs in CPR‐01 on chromosomes Ca1, Ca4 and Ca6, and six QTLs in CPR‐02 on chromosomes Ca2, Ca4 and Ca7. To validate the results, we compared the QTLs identified using NGS‐based BSA with the QTLs identified using conventional QTL mapping procedure. The CPR‐01 population was initially genotyped by RAD‐seq GBS approach resulting in the identification of eight QTLs for ascochyta blight resistance (Daba *et al*., 2016; Deokar *et al*., 2014). Five QTLs identified by NGS‐based BSA in CPR‐01 overlapped with the three QTLs previously reported in CPR‐01 using conventional QTL mapping (Table 1). The three overlapped QTLs are among the QTLs with moderate effects explaining 13, 17 and 19% phenotypic variation (PV), respectively, for ascochyta blight resistance in CPR01. The common and overlapped QTLs between the conventional and NGS‐based BSA approach in CPR‐01 population showed the reliability of NGS‐based BSA method to identify QTLs for ascochyta blight resistance in chickpea. However, two QTLs with relatively low PV (9% and 10%) and four QTLs with 12‐16% PV were undetected using the NGS‐based BSA analysis of CPR‐01 (Table 1). These QTLs were identified under specific environment or disease screening conditions such as either under greenhouse screening or under field screening in a specific year, whereas the common QTLs were among the QTLs consistently identified in multiple ascochyta blight screenings as reported by Daba *et al*. (2016). The detection power of NGS‐based BSA is positively correlated with the heritability, population size and sequencing depth (Guo *et al*., 2016). In case of CPR‐01, the small population size has likely resulted in lower power to detect QTLs with minor effects.

In CPR‐02 population, Amit is the source of ascochyta blight resistance and likely carries common, as well as different QTLs for resistance than the CDC Frontier, does in CPR‐01 population (Anbessa *et al*., 2009). Six QTLs were identified in CPR‐02, of which QTLs on chromosomes Ca2 and Ca7 are identified in this population. The QTL on chromosome Ca2 (CPR02‐qAB2.1) overlapped with one of the earlier reported QTLs (QTL1) (Anbessa *et al*., 2009), whereas the QTL on chromosome Ca7 (CPR02‐qAB7.1) is novel. The identification of novel QTLs for ascochyta blight will contribute to the understanding of the genetic architecture of the resistance to ascochyta blight in chickpea. The QTL CPR01‐qAB4.3 and CPR01‐qAB4.4 identified in CPR‐01 overlapped with a cluster of QTLs CPR02‐qAB4.1, CPR02‐qAB4.3 and CPR02‐qAB4.3 detected in CPR02. This showed that both CDC Frontier and Amit share common genomic regions on chromosome Ca4 for resistance to ascochyta blight.

Despite the different genetic backgrounds, pathogen structures and growing conditions, some QTLs were consistently reported in different studies (Anbessa *et al*., 2009; Daba *et al*., 2016; Flandez‐Galvez *et al*., 2003; Iruela *et al*., 2006). We also observed overlapping QTLs identified in CPR‐01 and CPR‐02 with some previously reported QTLs. Ascochyta blight resistance QTLs have been reported on all chickpea chromosomes. However, most of these QTLs were identified using low‐density SSR marker‐based maps and the QTL interval varied from 0.3 to 30 Mb in CDC Frontier physical map (Li *et al*., 2017; Sagi *et al*., 2017). Genomic position of a few QTL flanking SSR markers was still undetected in the reference genome, and, hence, they were not used for comparative analysis in the present study. The CPR02‐qAB2.1 QTL overlapped with a pathotype I‐specific QTL Ar19 and ar1 (Cho *et al*., 2004; Udupa and Baum, 2003), pathotype II‐specific QTLar2a (Udupa and Baum, 2003) and unknown pathotype‐associated QTL1 and QTL_AR3_ (Anbessa *et al*., 2009; Iruela *et al*., 2007). Both the pathotype I‐ and phenotype II‐specific loci on chromosome 2 were tightly linked (Udupa and Baum, 2003). All the reported QTLs in this region explained on an average 20% of the phenotypic variations for ascochyta blight resistance. Recently, using genetic and genomic tools this region has been narrowed down to 32‐ to 33‐Mb region which corresponds to 18–19 Mb in CDC Frontier genome assembly version Cav2 (Madrid *et al*., 2014). In our study, this locus was mapped as a single QTL in a 750‐kb region. This region may contain single or multiple genes that regulate individually or simultaneously the resistance to pathotypes I and II of *Ascochyta rabiei*. Therefore, introgression of this region into other chickpea cultivars might help to improve overall resistance against multiple pathotypes of *Ascochyta rabiei*. Similar observation of single QTL regulating resistance to multiple pathotypes has been reported in other species (Bilgic *et al*., 2006; Yu *et al*., 2017).

The CPR01‐qAB4.1 (1.7–8.9 Mb) overlapped with QTL_AR1_ (Cho *et al*., 2004; Iruela *et al*., 2006; Madrid *et al*., 2012). Furthermore, CaETR‐1, a homolog of Arabidopsis EIN4, was identified as a potential candidate gene in the QTL_AR1_ interval (4.4–8.0 Mb) (Madrid *et al*., 2012). CPR01‐qAB4.1 (1.7–8.9 Mb) and CPR01‐qAB4.2 (12.2–13.9 Mb) overlapped with a single QTL AB‐Q‐SR‐4‐1 (8.8–15.7 Mb) on chromosome 4 (Sabbavarapu *et al*., 2013). Recently, this genomic region has been narrowed down to 100 kb (15.8–15.9 Mb) using GWAS and fixation index (F_ST_) analysis (Li *et al*., 2017). However, this region falls outside of the significant QTL interval identified in CPR‐01. The G′ peak of the CPR01‐qAB4.1 and CPR01‐qAB4.2 was at 7.1 Mb and 13.3 Mb, respectively, which precisely co‐located with the earlier reported QTLs QTL_AR1_ (4.4–8.0 Mb) and QTL ar2b (15.6–22.3 Mb), respectively (Madrid *et al*., 2012; Udupa and Baum, 2003). It is possible that the region identified by Li *et al*. (2017) is different than the one identified in the current study as the two studies used different pathotypes of *Ascochyta rabiei* and different sources of resistance.

The NGS‐based BSA method identified a genomic region on chromosome Ca4 from 21.4 to 31.0 Mb that contains four common QTLs in both CPR‐01 and CPR‐02 populations (Figure 4). This region was also detected in the previous study as ar2b (Udupa and Baum, 2003), QTL_LG4 (Tar'an *et al*., 2007) and QTL_AR2_ (Iruela *et al*., 2006). A codominant SCAR marker (SCY17590) linked QTL_AR2_ has been used to trace the resistance alleles in 90% of resistant accessions in a collection of chickpea accessions from Spain, United States, Canada and ICARDA (Madrid *et al*., 2013).

In CPR‐01, two QTLs, QTL4 (5.4–53.8 Mb) and qtlAb‐6.1 (26.9–52.7 Mb), were mapped at the F_2_ (Tar'an *et al*., 2007) and the F_10_ (Daba *et al*., 2016) generations using conventional QTL mapping approach and, therefore, were highly expected to map the QTLs in the same genomic region using the NGS‐based BSA approach. However, the QTLs CPR01‐qAB6.1 (1.4–2.3 Mb) and CPR01‐qAB6.2 (62.9–64.2 Mb) did not overlap with the earlier reported QTL in CPR‐01, as these two QTLs mapped to the distinct ends of the chromosome Ca6 in the genome assembly v2.6. These two QTLs, however, were mapped at a single genomic location (10.2–17.2 Mb) based on the assembly version 1. This indicated some degrees of inconsistency in the genetic and physical order of the markers between the draft assemblies, and, therefore, none of our QTLs from CPR01 overlapped with the earlier reported QTLs on chromosome Ca6. Inconsistency in chickpea genome assemblies between different versions of draft genome and linkage maps had been pointed out earlier (Deokar *et al*., 2014; Parween *et al*., 2015; Ruperao *et al*., 2014).

The QTLs identified in this study with no overlapping to any of the previously reported QTLs are considered novel. The QTLs for resistance to ascochyta blight on chromosome Ca1 CPR01‐qAB1.2, CPR01‐qAB1.3 and CPR01‐qAB1.4 are novel. The QTL on chromosome Ca4 from CPR‐02 (qAB4.4; 54.2–55.5 Mb) which had the highest G′ value has also not been reported earlier. These novel QTLs have alleles from CDC Frontier or Amit that may enhance the resistance to ascochyta blight.

Genes with sequence variations between the resistant and susceptible lines that cause amino acid substitutions which can potentially result in moderate to high impact on gene function were identified within the significant interval of the mapped QTLs. Genes with homology to the earlier characterized genes involved in defence responses were selected as potential candidate genes (Table 3). Ethylene plays an important role in resistance to several necrotrophic pathogens (Berrocal‐Lobo *et al*., 2002; van Loon *et al*., 2006). Five ethylene‐responsive transcription factors (Ca07102, Ca07138, Ca11403, Ca11422 and Ca14698), two ethylene receptors ETR2 (Ca10965 and Ca12910) and one ethylene overproducing‐1‐like gene (Ca10695) that are involved in ET biosynthesis and signalling network were identified within the ascochyta blight QTL intervals (Table 3). Disease resistance genes, pathogenesis‐related genes transcriptional activator PTI5, pathogenesis‐related genes and multiple CC‐NBS‐LRR disease resistance genes were also identified in the QTL intervals (Table 3). Co‐localization of NBS‐LRR genes in QTL interval and differential expression profiles of NBS‐LRR genes in response to ascochyta blight infection on the resistant and susceptible chickpea cultivars suggested the possible involvement of NBS‐LRR genes in response to ascochyta blight in chickpea (Sagi *et al*., 2017). The interaction between flowering and resistance to diseases has been observed in multiple plants. The late‐flowering mutants of Arabidopsis show enhanced resistance to a hemibiotrophic pathogen *Fusarium oxysporum*, suggesting the relationship between flowering time and defence response in Arabidopsis (Lyons *et al*., 2015). A negative correlation between flowering and ascochyta blight resistance and co‐localization of QTLs for flowering and ascochyta blight have been reported earlier in chickpea (Daba *et al*., 2016). This relationship could be due to either pleiotropic effect of flowering genes on disease resistance or linkage of some flowering genes with the QTL governing resistance to ascochyta blight. In the present study, we also identified two flowering genes, photoperiod‐independent early flowering 1 (Ca13027) and flowering locus D (Ca14012), within the ascochyta blight resistant QTLs CPR01‐qAB4.4, CPR01‐qAB4.5, and CPR02‐qAB4.2. Further analysis is needed to understand the relationship between flowering and ascochyta blight resistance. The large QTL interval (few Mb in size) of the QTLs detected in present study limits the precise prediction of the candidate genes; however, the SNPs identified in the QTL regions can be used to narrow down the QTL interval.

**Table 3:** List of potential candidate genes for ascochyta blight resistance underlying the QTL for ascochyta blight resistance identified in CPR‐01 and CPR‐02 populations

Chr^a^.	SNP/InDel position^a^	SNP/InDel	Amino acid changes	Gene code^a^	Start^a^	End^a^	Annotations^a^
CPR01‐qAB1.1							
Ca1	2001205	A/AT	Leu140fs	Ca07030	2001002	2002378	Secretory carrier‐associated membrane protein
Ca1	2617844	T/G	Thr92Pro	Ca07102	2617455	2618117	Ethylene‐responsive transcription factor 4
Ca1	2727738	T/A	Ser558Thr	Ca07109	2726067	2729458	Receptor‐like protein kinase HSL1
Ca1	2827727	A/G	Ser958Gly	Ca07110	2823270	2828262	Putative disease resistance protein At3g14460
Ca1	2745915	A/C	Gln103Pro	Ca07115	2745476	2746047	NAC domain‐containing protein 73‐like
Ca1	2748196	G/A	Gly130Ser	Ca07116	2747795	2748339	NAC domain‐containing protein 73‐like
Ca1	2820081	A/G	Lys32Arg	Ca07117	2819987	2823109	Putative disease resistance protein At3g14460
Ca1	2750431	C/A	Arg320Ser	Ca07119	2749474	2751120	Putative disease resistance protein At3g14460
Ca1	2702329	C/G	Gly482Arg	Ca07126	2699783	2703772	CC‐NBS‐LRR disease resistance protein
Ca1	2798214	T/G	Ser553Arg	Ca07127	2797891	2801614	CC‐NBS‐LRR disease resistance protein
Ca1	2889473	A/C	Gln44His	Ca07132	2889342	2889854	Pathogenesis‐related genes transcriptional activator PTI5
Ca1	2894124	T/C	Tyr194Cys	Ca07138	2893922	2894704	Ethylene‐responsive transcription factor 1B
Ca1	3082310	A/G	Gln261Arg	Ca07147	3081101	3082586	Cysteine protease
Ca1	3062221	G/A	Ser387Leu	Ca07156	3061836	3067613	Protein trichome birefringence‐like 38
Ca1	4641168	A/T	Val284Asp	Ca07320	4639199	4642642	Receptor‐like cytosolic serine/threonine‐protein kinase
CPR01‐qAB1.3							
Ca1	30861100	G/T	Ala372Glu	Ca09427	30845621	30865458	Glucan synthase‐like protein
Ca1	30981183	T/C	His517Arg	Ca09434	30978636	30983325	TMV resistance protein N‐like isoform X1
CPR01‐qAB1.4							
Ca1	35059453	T/C	Asn48Ser	Ca09699	35058791	35059699	Protein trichome birefringence‐like 33
Ca1	35472581	T/C	Phe262Ser	Ca09717	35471404	35472816	NAC domain‐containing protein 76
Ca1	36156793	A/G	Asn300Ser	Ca09757	36155895	36159695	Protein ASPARTIC PROTEASE IN GUARD CELL 1
Ca1	36800678	A/C	Phe98Val	Ca09855	36800175	36801119	Chitinase 2‐like
CPR02‐qAB2.1							
Ca2	18232910	A/G	Ile119Met	Ca30034	18230538	18233366	Ankyrin repeat protein
Ca2	18250143	T/A	Gln1075His	Ca30037	18249996	18256970	TMV resistance protein
Ca2	18309282	C/T	Ser699Leu	Ca30040	18307187	18310122	LRR receptor‐like serine/threonine‐protein kinase
Ca2	18266481	A/C	Cys75Gly	Ca30038	18260228	18266703	TMV resistance protein N‐like
CPR01‐qAB4.1							
Ca4	1747100	T/C	Ile14Thr	Ca10690	1747060	1749119	Inhibitor of apoptosis‐promoting Bax1 protein
Ca4	1732780	A/T	Phe1009Leu	Ca10695	1732724	1737719	ETO1‐like protein 1 isoform X1
Ca4	4413663	A/G	Asp587Gly	Ca10965	4411823	4415096	Ethylene receptor ETR2
Ca4	4560351	A/T	Asn454Ile	Ca10975	4558991	4560592	Ankyrin repeat domain‐containing protein 13C
Ca4	4656331	A/T	Ile1011Lys	Ca10992	4656297	4659452	Leucine‐rich repeat receptor‐like protein kinase PXL1
Ca4	4807344	T/C	Gln55Arg	Ca11021	4806618	4807507	Pathogenesis‐related protein PR‐4‐like
Ca4	6698231	A/C	Lys16Thr	Ca11238	6698185	6699834	Autophagy‐related protein
Ca4	7942281	G/T	Lys257Asn	Ca11378	7941511	7943415	LRR receptor‐like serine/threonine‐protein kinase RKF3
Ca4	7906124	T/G	Thr88Pro	Ca11382	7905207	7906385	LRR receptor‐like serine/threonine‐protein kinase ERL1
Ca4	7924151	C/A	Gly184^a^	Ca11385	7918963	7924946	Autophagy‐related protein 18g‐like
Ca4	8135769	T/C	Ser337Pro	Ca11403	8134761	8136005	Ethylene‐responsive transcription factor ERF062
Ca4	8094507	A/T	Asn185Lys	Ca11409	8090053	8095218	Probable glutathione S‐transferase isoform X1
Ca4	8188167	C/G	Glu24Asp	Ca11422	8187627	8188238	Ethylene‐responsive transcription factor ERF107
Ca4	8383716	A/G	Val404Ala	Ca11438	8380604	8384926	CC‐NBS‐LRR disease resistance protein
Ca4	8398595	A/G	Asn1005Asp	Ca11441	8395583	8399902	NBS‐LRR protein
Ca4	8375578	A/T	Gln1152His	Ca11446	8371918	8376295	Putative disease resistance RPP13‐like protein 2
Ca4	8466370	T/C	Ser263Pro	Ca11448	8465016	8469257	NAC domain‐containing protein 7
Ca4	8395888	C/T	Arg91Gln	Ca11455	8395835	8401268	NBS‐LRR protein
CPR01‐qAB4.2							
Ca4	12213783	A/G	Ser522Gly	Ca11808	12206425	12214215	F‐box/LRR‐repeat protein At3g48880
Ca4	13169125	C/G	Ala71Gly	Ca11887	13168914	13171681	Nonspecific lipid transfer protein GPI‐anchored 2‐like
CPR01‐qAB4.3							
Ca4	21779099	A/G	Tyr153His	Ca12602	21778761	21779690	Vegetative cell wall protein gp1‐like
Ca4	21829784	T/G	Ser307Ala	Ca12606	21828866	21833474	Calcium‐dependent protein kinase 20
Ca4	22237672	T/C	Asp203Gly	Ca12638	22235653	22238279	Syntaxin of plants 122 protein
CPR01‐qAB4.4, CPR02‐qAB4.1, CPR02‐qAB4.2, CPR02‐qAB4.3							
Ca4	24761966	C/A	Ser401Ile	Ca12806	24761323	24763167	Ankyrin repeat domain‐containing protein 13C‐like
Ca4	25036762	C/T	Ser402Phe	Ca12823	25035445	25037121	Vegetative cell wall protein gp1
Ca4	25315050	T/C	Ile52Val	Ca12840	25311054	25315203	Aspartic protease
Ca4	26669292	T/G	Ile157Arg	Ca12910	26668823	26671205	Ethylene receptor 2‐like
Ca4	27339681	T/G	Thr232Pro	Ca12943	27339520	27340379	Leucine‐rich repeat extensin‐like protein 5
Ca4	27427312	T/C	Leu503Pro	Ca12945	27421878	27427957	Casein kinase I‐like protein
Ca4	28227316	T/C	Ile131Thr	Ca12987	28226925	28229770	Protein trichome birefringence‐like
Ca4	28791114	G/C	Pro984Ala	Ca13027	28784513	28807072	Photoperiod‐independent early flowering 1
Ca4	31114113	T/C	Gln85Arg	Ca13180	31110361	31114366	Vegetative cell wall protein gp1‐like
CPR01‐qAB4.5							
Ca4	43775108	C/G	Leu84Val	Ca14001	43774492	43776344	Universal stress protein A
Ca4	43806808	A/G	Ile26Val	Ca14012	43806733	43812564	Protein flowering locus D
CPR02‐qAB4.4							
Ca4	55177211	G/T	Thr13Lys	Ca14675	55174708	55177248	Putative B3 domain‐containing protein At1g78640
Ca4	55472030	C/T	Glu391Lys	Ca14698	55471413	55475414	AP2‐like ethylene‐responsive transcription factor ANT
CPR01‐qAB6.1							
Ca6	63248069	G/A	Gly49Asp	Ca06534	63247754	63248160	Receptor‐like kinase
Ca6	63246080	A/T	Tyr144Asn	Ca06542	63245150	63247800	Receptor‐like kinase
Ca6	63125250	A/G	Val325Ala	Ca06547	63123212	63126223	Probable LRR receptor‐like serine/threonine‐protein kinase
Ca6	63396151	A/T	Glu6Val	Ca06589	63396135	63400440	Glucan endo‐1,3‐beta‐glucosidase 4
Ca6	63549230	A/C	Phe19Cys	Ca06606	63548387	63549285	Plasmodesmata callose‐binding protein 3‐like
Ca6	63570255	G/C	Ser377Cys	Ca06628	63568134	63590605	Plasmodesmata callose‐binding protein 3
Ca6	63712079	G/T	His20Asn	Ca06632	63710609	63712136	Chalcone synthase 1
Ca6	63898200	G/A	Thr14Ile	Ca06642	63894526	63898240	Receptor protein kinase TMK1

a Genomic position and gene annotations are based on the CDC Frontier reference genome assembly v2.6.3.

Nonsynonymous SNPs and the corresponding amino acid changes in the candidate genes are shown.

In addition to the identification of potential candidate genes within the QTL intervals, allele‐specific SNP markers (KASP™ assays) for six candidate genes within the QTL interval on chromosomes Ca2 and Ca4 were developed for the validation study. Single marker analysis of variance (ANOVA) showed significant marker‐trait association for all the selected candidate genes. Overall, this study confirmed the efficiency of NGS‐based BSA as a rapid and cost‐effective method to identify QTLs associated with ascochyta blight in chickpea.

## Experimental procedures

### Ascochyta blight screening

Two chickpea RIL populations (CPR‐01 and CPR‐02) were used to identify the genomic regions associated with the resistance to ascochyta blight. CPR‐01 contains 92 RILs (F_10_) derived from a cross between ICCV 96029 (ascochyta blight susceptible) and CDC Frontier (ascochyta blight moderate resistant). CPR‐02 contains 139 RILs (F_10_) derived from a cross between ICCV 96029 and Amit (ascochyta blight moderate resistant). Both RIL populations along with the parents (ICCV 96029, CDC Frontier and Amit) were screened for response to ascochyta blight under controlled (greenhouse) and field conditions. In the greenhouse, one plant was grown per 10 cm^2^ pot. The day/night temperature of 20/16 °C and photoperiod of 16 h were maintained using artificial light sources. Both populations were evaluated in three replications arranged in a completely randomized design. The entire experiment was repeated twice. Three‐week‐old seedlings at the 10‐internode stage were inoculated with *Ascochyta rabiei* isolate AR170‐3 as described in Anbessa *et al*. (2009) and Tar'an *et al*. (2007). The disease response was evaluated 2 weeks after inoculation using 0‐9 scale (0 = no symptoms, 9 = susceptible) (Reddy and Singh, 1984).

The RIL populations and the parental genotypes were also screened for ascochyta blight responses under field conditions at multiple locations (Elrose, Limerick and Moose Jaw) in Saskatchewan, Canada, between the years 2011 and 2015. The CPR‐01 was screened in the years 2011, 2012 and 2013 at two locations per year (Daba *et al*., 2016). The CPR‐02 was screened at Elrose, Saskatchewan, in 2014 and 2015 growing seasons. Each population during the field screening was arranged in a completely randomized block design with three replications. Each RIL was planted in the three‐row plot of 1 m × 1 m size. Disease rating was carried out based on the overall disease development within a plot at the early podding stage using a 0–9 rating scale.

### Selection of RILs for resistant and susceptible bulks

Selection of the RILs with the extreme response to *Ascochyta rabiei* infection was based on the frequency distribution of disease reaction, mean and standard deviation from the individual field and greenhouse screenings. The response to ascochyta blight is dependent on plant development stage and the environmental conditions in the field as well as the growth conditions in the greenhouse. Therefore, instead of selecting the RILs with specific disease response scores as cut‐off values, we selected the RILs exhibiting extreme disease response using a two‐step selection process. First, in individual screening, RILs showing disease scores one standard deviation below or above the population mean score were selected. Second, the RILs with consistent extreme scores in multiple screenings were selected. In CPR‐01, ten RILs were selected and used to prepare each of the resistant and susceptible bulks. For CPR‐02, 14 RILs were selected to form each of resistant and susceptible bulks.

### Whole‐genome sequencing of ICCV 96029, Amit and bulked segregants

Genomic DNA was isolated from the selected RILs individually using CTAB method and further purified using Qiagen DNeasy Plant Mini Kit. DNA quality was evaluated by running the samples on 1% agarose gel and was quantified using NanoDrop^®^. DNA concentration was adjusted to a final concentration of 100 ng/μL, and 1 μg of total DNA from each RIL was used for bulking. Two DNA pools of one for each of resistant and susceptible bulks were generated by equally mixing 10 individual DNAs from each group for CPR‐01 and 14 individuals for CPR‐02. The pooled DNA samples were sequenced at the sequencing facility of the Beijing Genomics Institute (BGI) using the Illumina's HiSeq X sequencing system. Parental genotypes ICCV 96029 and Amit were sequenced on Illumina HiSeqTM 2000.

### Generation of pseudo‐reference genome of ICCV 96029

Raw reads were processed using Trimmomatic (v 0.35) to remove low‐quality sequencing reads and any adapter contamination. The clean reads were mapped onto CDC Frontier v2.6.3 reference genome (Edwards, 2016: https://doi.org/10.7946/p2g596) using Burrows‐Wheeler Aligner (BWA) MEM. Picard Tools ‘SortSam’ was used to sort mapped reads. ‘MarkDuplicates’ was used to locate duplicate molecules and ‘BuildBamIndex’ to index the BAM files with the default parameters (http://broadinstitute.github.io/picard). SNPs and InDels were called using GATK (v.3.7) ‘HaplotypeCaller’ (DePristo *et al*., 2011; Van der Auwera *et al*., 2013). Only the SNPs were used to construct ICCV 96029 pseudo‐genome using GATK's tool ‘FastaAlternateReferenceMaker’ which generates an alternative reference sequence by replacing the reference bases at site variations with the alternate bases supplied in the corresponding callset records (Van der Auwera *et al*., 2013).

### NGS‐based BSA analysis

The clean reads from the resistant and susceptible bulks of CPR‐01 and CPR‐02 were mapped onto the CDC Frontier and pseudo‐reference genome assembly of ICCV 96029, respectively, and then, the variants were called. SNPs with reference allele frequency of 0.2 in both bulks were filtered out as these may due to sequencing or alignment errors. NGS‐based BSA analysis was performed following the earlier reports (Magwene *et al*., 2011; Takagi *et al*., 2013; Yang *et al*., 2013). Briefly, SNP index was calculated as the ratio of the alternative allele reads to the total read depth in the individual bulk samples (resistant and susceptible bulks). Then, delta SNP index was calculated by subtracting the SNP index of the resistance bulk from the SNP index of the susceptible bulk (Takagi *et al*., 2013). The genome‐wide G statistics (G value) for each SNP was calculated by observed and expected allele depth of the reference and alternate allele assuming that the allele depth is equal for both resistance and susceptible bulks. G′ value was calculated using a tricube‐smoothed G value by average weighted of the physical distance across the neighbouring SNPs within a given window that accounts for Linkage disequilibrium (LD) and also minimize noise attributed to SNP calling errors (Magwene *et al*., 2011; Yang *et al*., 2013). *P*‐values are estimated using a nonparametric estimation of the null distribution of the G′ values assuming no QTL (Magwene *et al*., 2011). Recently, Mansfeld and Grumet (2018) described a method that uses delta SNP values instead of Hampel's rule to estimate the null distribution parameters. We used delta SNP index (0.1) to calculate *P*‐values and a FDR (q) of 0.001 to identify potential QTLs associated with ascochyta blight resistance. The NGS‐based BSA analysis method described above and used in this analysis is available in a R package QTLseqr (https://github.com/bmansfeld/QTLseqr) developed by Mansfeld and Grumet (2018).

### Candidate genes

SNPs identified between the resistant and susceptible parental lines and within the QTL regions were subjected to the annotation to detect the effect of these SNPs using Snpeff (Cingolani *et al*., 2012). Homologs of the earlier characterized genes involved in disease response or responsive to biotic stresses and having SNPs with high to moderate variant impacts were selected as potential candidate genes involved in ascochyta blight resistance in chickpea.

### Development of competitive allele‐specific genotyping assays and genotyping CPR‐02 RIL population

SNP information from the candidate genes within the QTL interval was used to design allele‐specific PCR genotyping assays using the Kompetitive Allele‐Specific PCR genotyping system (KASP) (LGC Genomics). For each SNP, two allele‐specific primers and one common primer were designed using primer picker software. Genomic DNA from Amit, ICCV 96029 and the CPR‐02 RILs was isolated using CTAB method and normalized to 10 ng/μL. KASP™ genotyping reaction was performed using Bio‐Rad CFX Connect™ Real‐Time PCR Detection System. KASP analysis was performed following the protocol described by Thompson and Tar'an (2014).

### QTL analysis using single marker analysis of variance (ANOVA)

We used single marker ANOVA to map the QTLs. Briefly, for each of the SNP marker, the RILs were grouped according to the SNP allele, and then, one way ANOVA was used to test for significant difference between the group means. F‐statistics in the ANOVA was used to define significant marker‐QTL association. We used *P*‐value <0.05 to define significant marker‐QTL association. R‐Square value from the ANOVA analysis was interpreted as a measurement of the proportion of phenotypic variation explained by the QTL.

## Author's contributions

A.D. performed the experiments; A.D., M.S and K.D performed disease screenings of the RILs. A.D. performed NGS‐based BSA analysis; A.D. and M.S. performed candidate SNP validation experiment; A.D, M.S, K.D and B.T. wrote the manuscript; AD and B.T. conceived, designed and supervised the research and finalized the manuscript. All authors read and approved the manuscript.

## Conflict of interest

The author(s) declare that they have no competing interests.

## Supporting information


**Figure S1** Frequency distribution ascochyta blight disease scores in 92 RILs of CPR‐01 population developed from a cross between ICCV 96029 and CDC Frontier.

**Table S1** Summary of analysis of variance for ascochyta blight scores in the CPR‐02 RILs tested under greenhouse (combined data of two experimental repeats of three replications each) and field (combined and individual data from field conditions at Elrose, Saskatchewan, Canada in 2014 and 2015).
**Table S2** Summary of QTLs for resistance to ascochyta blight in two recombinant inbred populations of chickpea (CPR‐01 and CPR‐02) identified using the NGS‐based BSA approach.
**Table S3** Primer sequences of KASP™ assays used in the validation study.
