# Supplementary material for: QTL sequencing strategy to map genomic regions associated with resistance to ascochyta blight in chickpea
Source: Plant Biotechnol J. 2018 Jul 4;17(1):275–88. doi: 10.1111/pbi.12964 (PMC6330535; doi:10.1111/pbi.12964)
Supplement: Supplementary file 1 — Figure S1 Frequency distribution ascochyta blight disease scores in 92 RILs of CPR‐01 population developed from a cross between ICCV 96029 and CDC Frontier. [file PBI-17-275-s001.docx]

**Ascochyta blight disease score**

**Number of RILs**


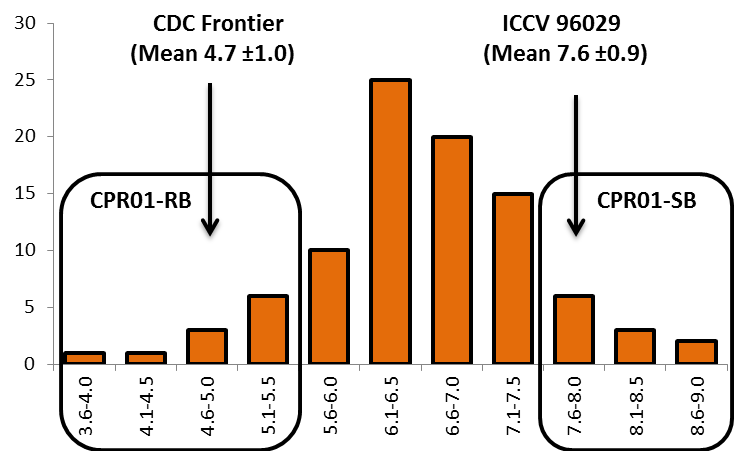


**a**

**b**

**Ascochyta blight disease score**

**Number of RILs**


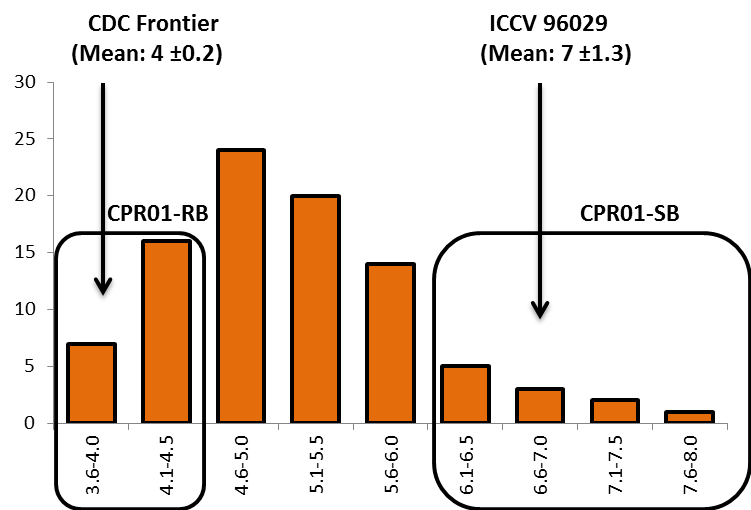


**Ascochyta blight disease score**

**Number of RILs**


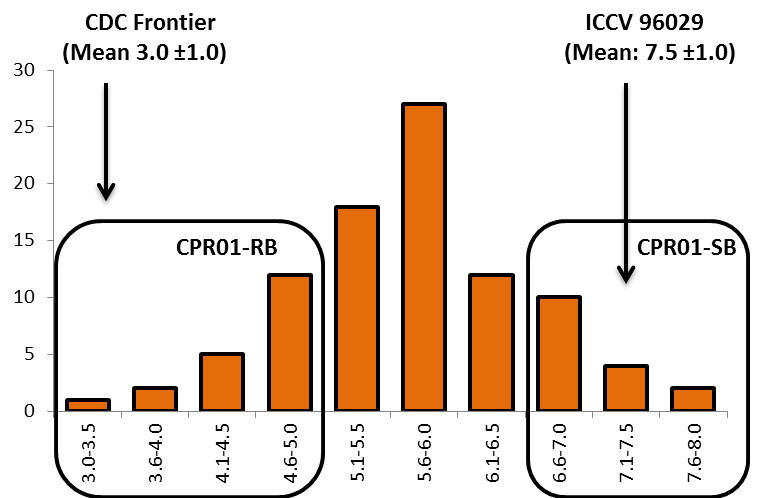


**c**

**d**

**Ascochyta blight disease score**

**Number of RILs**


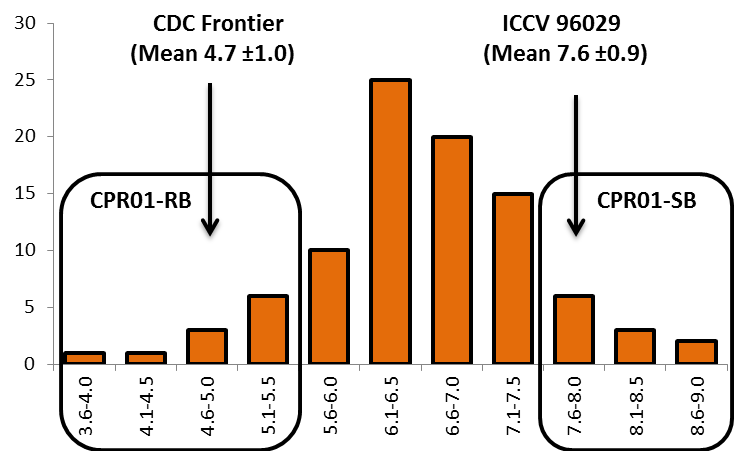


**Figure S1: Frequency distribution ascochyta blight disease scores in 92 RILs of CPR-01 population developed from a cross between ICCV 96029 and CDC Frontier.** Mean disease score was used to calculate the frequency distribution of disease scores in the greenhouse experiment repeats (a), field conditions at Elrose 2011, 2012 and 2013 (b), Limerick 2013 (c) and Moose Jaw 2012 (d). Arrows show the mean scores of the resistant (Amit) and the susceptible (ICCV 96029) parents. RILs exhibiting extreme disease response on both ends of the scale were selected to construct resistant (CPR01-RB) and susceptible (CPR01-SB) bulks.
