# Supplementary material for: QTL sequencing strategy to map genomic regions associated with resistance to ascochyta blight in chickpea
Source: Plant Biotechnol J. 2018 Jul 4;17(1):275–88. doi: 10.1111/pbi.12964 (PMC6330535; doi:10.1111/pbi.12964)
Supplement: Supplementary file 2 — Table S1 Summary of analysis of variance for ascochyta blight scores in the CPR‐02 RILs tested under greenhouse (combined data of two experimental repeats of three replications each) and field (combined and individual data from field conditions at Elrose, Saskatchewan, Canada in 2014 and 2015). Table S2 Summary of QTLs for resistance to ascochyta blight in two recombinant inbred populations of chickpea (CPR‐01 and CPR‐02) identified using the NGS‐based BSA approach. Table S3 Primer sequences of KASP™ assays used in the validation study. [file PBI-17-275-s002.docx]

**Table S1:** Summary of analysis of variance for ascochyta blight scores in the CPR-02 RILs evaluated under greenhouse (combined data of two experimental repeats of three replications each) and field (combined and individual data from field conditions at Elrose, Saskatchewan, Canada, in 2014 and 2015).

| **Location** | **Level** |  | **Effect** | **CPR-02** |
| --- | --- | --- | --- | --- |
| Greenhouse | Combined |  | G | 7.14*** |
|  |  |  | R | 0.48^ns^ |
|  |  |  | G x R | 0.36^ns^ |
|  |  |  | *σ^2^_G_* | 0.77 |
|  |  |  | *σ^2^_GR_* | 0.00 |
|  |  |  | *σ^2^_er_* | 0.52 |
|  |  |  | *H^2^* | 0.59 |
| Elrose | Combined |  | G | 8.31*** |
|  |  |  | Y | 75.86*** |
|  |  |  | G x Y | 2.74*** |
|  |  |  | *σ^2^_G_* | 0.36 |
|  |  |  | *σ^2^_GY_* | 0.16 |
|  |  |  | *σ^2^_er_* | 0.45 |
|  |  |  | *H^2^* | 0.37 |
| Elrose | 2014 |  | G | 5.05*** |
|  |  |  | *σ^2^_G_* | 0.59 |
|  |  |  | *σ^2^_er_* | 0.30 |
|  |  |  | *H^2^* | 0.65 |
| Elrose | 2015 |  | G | 6.43*** |
|  |  |  | *σ^2^_G_* | 0.49 |
|  |  |  | *σ^2^_er_* | 0.52 |
|  |  |  | *H^2^* | 0.48 |

Note: G= Genotype which representing each inbred line, R= Repeat, Y=Year, G x R= Genotype by repeat interaction, G x Y= Genotype by year interaction, and *σ^2^_G,_ σ^2^_GR_, σ^2^_GY,_* and *σ^2^_er_* = estimate of genotypic, genotype by repeat, genotype by year and error variance, *H^2^* is broad sense heritability, and ‘*’, ‘**’ and ‘***’ indicates significant difference at *P* ≤0.05, 0.01, 0.001 and ns=non-significant.

**Table S2:** Summary of QTLs for resistance to ascochyta blight in two recombinant inbred populations of chickpea (CPR-01 and CPR-02) identified using the NGS-based BSA approach.

| **QTL Name** | **Chr.^#^** | **start^#^** | **end^#^** | | **QTL interval ^#^ (Mb)** | **Max G’ value** | **Mean**  **P-value** | **Trait contributing allele** |
| --- | --- | --- | --- | --- | --- | --- | --- | --- |
| **CPR-01 (ICCV96029 X CDC Frontier)** | | | | | | | | |
| CPR01-qAB1.1 | Ca1 | 1,999,318 | 5,345,307 | | 3.35 | 12.88 | 4.81E-07 | CDC Frontier |
| CPR01-qAB1.2 | Ca1 | 10,611,232 | 12,383,822 | | 1.77 | 9.28 | 8.91E-07 | ICCV 96029 |
| CPR01-qAB1.3 | Ca1 | 30,179,115 | 31,355,738 | | 1.18 | 5.68 | 7.57E-05 | CDC Frontier |
| CPR01-qAB1.4 | Ca1 | 33,954,240 | 36,806,552 | | 2.85 | 6.74 | 1.35E-05 | CDC Frontier |
| CPR01-qAB4.1 | Ca4 | 1,705,617 | 8,927,881 | | 7.22 | 16.32 | 1.73E-07 | CDC Frontier |
| CPR01-qAB4.2 | Ca4 | 12,218,355 | 13,965,172 | | 1.75 | 10.36 | 1.38E-05 | CDC Frontier |
| CPR01-qAB4.3 | Ca4 | 21,308,168 | 22,718,853 | | 1.41 | 6.16 | 3.54E-05 | CDC Frontier |
| CPR01-qAB4.4 | Ca4 | 24,079,492 | 31,119,455 | | 7.04 | 6.50 | 3.65E-05 | CDC Frontier |
| CPR01-qAB4.5 | Ca4 | 43,767,782 | 45,358,899 | | 1.59 | 5.73 | 6.59E-05 | CDC Frontier |
| CPR01-qAB6.1 | Ca6 | 1,463,739 | 2,329,958 | | 0.87 | 6.04 | 4.04E-05 | CDC Frontier |
| CPR01-qAB6.2 | Ca6 | 62,932,215 | 64,247,506 | | 1.32 | 10.07 | 1.12E-07 | CDC Frontier |
| **CPR-02 (ICCV96029 X Amit)** | | | | | | | | |
| *CPR02-qAB2.1* | Ca2 | 18,172,683 | | 18,924,423 | 0.75 | 6.46 | 1.23E-05 | Amit |
| *CPR02-qAB4.1* | Ca4 | 24,385,212 | | 27,090,913 | 2.71 | 6.56 | 1.10E-05 | Amit |
| *CPR02-qAB4.2* | Ca4 | 27,811,455 | | 28,234,398 | 0.42 | 5.73 | 2.82E-05 | Amit |
| *CPR02-qAB4.3* | Ca4 | 28,763,335 | | 31,068,540 | 2.31 | 6.83 | 6.09E-06 | Amit |
| *CPR02-qAB4.4* | Ca4 | 54,159,134 | | 55,479,633 | 1.32 | 9.30 | 3.64E-07 | Amit |
| *CPR02-qAB7.1* | Ca7 | 48,740,698 | | 48,980,912 | 0.24 | 6.30 | 6.50E-06 | Amit |

^#^ based on CDC Frontier genome assembly version 2.6.3

Resistant parent CDC Frontier and Amit; susceptible parent ICCV 96029

**Table S3**: Primer sequences of KASP™ assays used in the validation study.

| **Sr. No/Primer information** | **Marker Name/Primer name** | **Primer sequence (5'-3')** |
| --- | --- | --- |
| 1 | Ca2v2.6p18233152_G/A |  |
| Allele1 primer | Ca2v2.6p18233152_G | GAAGGTGACCAAGTTCATGCTCAAAGGTGTATTTCCTTCTTTATCCC |
| Allele2 primer | Ca2v2.6p18233152_A | GAAGGTCGGAGTCAACGGATTGCAAAGGTGTATTTCCTTCTTTATCCT |
| Common primer | Ca2v2.6p18233152_C1 | ATATTACAAAGCCAAACTCCTGAACTTCAT |
| 2 | Ca2v2T_Ap18250143_A/T |  |
| Allele1 primer | Ca2v2T_Ap18250143_A | GAAGGTCGGAGTCAACGGATTAAGTGGAGGTAAAGACCAACTTCAT |
| Allele2 primer | Ca2v2T_Ap18250143_T | GAAGGTGACCAAGTTCATGCTAAGTGGAGGTAAAGACCAACTTCAA |
| Common primer | Ca2v2T_Ap18250143_C1 | CCCACATTTGGTTATTGATGCTTTCCAAT |
| 3 | Ca2v2.6p18266481_A/C |  |
| Allele1 primer | Ca2v2.6p18266481_A | GAAGGTGACCAAGTTCATGCTTCTAGTTCATCCAAACGCAAAGTACA |
| Allele2 primer | Ca2v2.6p18266481_C | GAAGGTCGGAGTCAACGGATTCTAGTTCATCCAAACGCAAAGTACC |
| Common primer | Ca2v2.6p18266481_C1 | AAAAGTCCCTCGCTGCAATTGTAGTATT |
| 4 | Ca2v2.6p28572458_G/A |  |
| Allele1 primer | Ca2v2.6p28572458_G | GAAGGTGACCAAGTTCATGCTGGTCGAGCCATCAGACAAATCG |
| Allele2 primer | Ca2v2.6p28572458_A | GAAGGTCGGAGTCAACGGATTAAAGGTCGAGCCATCAGACAAATCA |
| Common primer | Ca2v2.6p28572458_C1 | TCAGTCATTAACTTTGCCACAGCAGAAAT |
| 5 | Ca4v2T_Gp26669292_G/T |  |
| Allele1 primer | Ca4v2T_Gp26669292_G | GAAGGTCGGAGTCAACGGATTCATTTTTCTTCATTATCATACCAACTTCTC |
| Allele2 primer | Ca4v2T_Gp26669292_T | GAAGGTGACCAAGTTCATGCTTCATTTTTCTTCATTATCATACCAACTTCTA |
| Common primer | Ca4v2T_Gp26669292_C1 | GTTAAGCTCAGAGAGTTTTTGTTGAAGATT |
| 6 | Ca4v2G_Cp28791114_C/G |  |
| Allele1 primer | Ca4v2G_Cp28791114_C | GAAGGTCGGAGTCAACGGATTCGTTCCATAATTGAAGTTGCAGGC |
| Allele2 primer | Ca4v2G_Cp28791114_G | GAAGGTGACCAAGTTCATGCTCGTTCCATAATTGAAGTTGCAGGG |
| Common primer | Ca4v2G_Cp28791114_C1 | TATAGCATGACTTCTTGGGAGAGTGAT |
| 7 | Ca4v2.6p43806808_A/G |  |
| Allele1 primer | Ca4v2.6p43806808_A | GAAGGTGACCAAGTTCATGCTCCAAACCGCATCAACTGCCTC |
| Allele2 primer | Ca4v2.6p43806808_G | GAAGGTCGGAGTCAACGGATTCCCAAACCGCATCAACTGCCTT |
| Common primer | Ca4v2.6p43806808_C1 | GGTGTGATTATAATTGGTGCTGGCCT |
| 8 | Ca4v2.6p 904185_A/C |  |
| Allele1 primer | Ca4v2.6p 904185_A | GAAGGTGACCAAGTTCATGCTTTGATGATAATGCTGCATACCGCA |
| Allele2 primer | Ca4v2.6p 904185_C | GAAGGTCGGAGTCAACGGATTTGATGATAATGCTGCATACCGCC |
| Common primer | Ca4v2.6p 904185_C1 | CCTCTTGTGTTGTATCACGAAGTGCAA |
